# Measurements of Temperature and Humidity Responsive Swelling of Thin Hydrogel Films by Interferometry in an Environmental Chamber

**DOI:** 10.3390/polym14193987

**Published:** 2022-09-23

**Authors:** Katrin Unger, Marlene Anzengruber, Anna Maria Coclite

**Affiliations:** Institute of Solid State Physics, NAWI Graz, Graz University of Technology, Petersgasse 16, 8010 Graz, Austria

**Keywords:** multi-responsive hydrogel, measurement technique, environmental parameters, hydrogel thickness, poly(N vinyl caprolactam), smart hydrogel, interferometry

## Abstract

Thin film thermo-responsive hydrogels have become a huge interest in applications such as smart drug-delivery systems or sensor/actuator technology. So far, mostly, the response of such hydrogels has been measured only by varying the temperature in a liquid environment, but studies of the response towards humidity and temperature are rare because of experimental limitations. Often the swelling measurements are performed on samples placed on a stage that can be heated/cooled, while vapors enter the permeation chamber at their own temperature. This thermal difference leads to some uncertainties on the exact relative humidity to which the sample is exposed to. In this study, we explored the possibility of performing swelling measurements under thermal equilibrium by placing the sample and an interferometer, as a detector, in an environmental chamber and therefore exposing the smart hydrogel to adjustable temperatures and relative humidity conditions while measuring the hydrogel’s thin film thickness changes. As a case study, we used thin films of the thermo-responsive hydrogel, poly N-vinylcaprolactam deposited by initiated chemical vapor deposition (iCVD). Similar thin films were previously characterized by in situ ellipsometry while the sample was heated on a stage and exposed to humid air produced at room temperature. The comparison between the two measurement methods showed that while measurements in the presence of thermal gradients are limited mostly to low humidity, measurements in thermal equilibrium are restricted only by the operation limits of the used environmental chamber.

## 1. Introduction

Smart hydrogels are water-swollen polymer networks that respond to an external trigger, such as temperature, light, pH value, or chemical analytes, with a change of magnitude in swelling, wettability, optical parameter, or elasticity [1,2]. In certain applications, such as smart drug encapsulation or sensor/actuator technologies, a thin film of such material is required [3,4,5]. The implementation of a thin film smart hydrogel in such a delicate usage requires well-known insight into the material’s response towards the environmental parameters. The characterization of multi-responsive thin films reaction to two parameters remains challenging and requires specific and often expensive instrument setups.

So far, studies on the thickness of thermo-responsive hydrogel’s thin films versus temperature and relative humidity were performed when the sample was placed on a heating plate while the humidified air was supplied at room temperature [6,7,8,9]. Under such conditions, the relative humidity directly at the sample surface is not the same as the relative humidity of the environment.

According to the Flory-Rehner theory, the thickness change of cross-linked hydrogels is dependent on the samples’ relative humidity and the Flory Huggins polymer–vapor interaction parameter [10,11,12]. Importantly, the dependency is on the relative humidity and not on the water vapor weight the air carries. If the sample is heated, the supplied humidified air will not contain enough water vapour amount to reach higher relative humidity levels at the hot sample. Hence, the currently used measurement technique does not allow one to access the whole relative humidity–temperature parameter space.

Additionally, when the sample is cooler than the supplied air, the prevention of condensation needs to be considered. The supplied relative humidity needs to be chosen wisely to not reach conditions in which the sample temperature is below the dew point. The dew point is the temperature value to which air needs to be cooled to reach water vapour saturation, and it is dependent on the relative humidity and the temperature of the air. A typical phenomenon is the fogging of glasses when moving from the cold outside into the warm inside. 

In addition, temperature gradients can hinder a correct study and an interpretation of the response of thermo-responsive materials in a humid environment. In a system where humidity flows at room temperature and the stage is heated, a reliable characterization of thermo-responsiveness can be conducted by keeping the temperature constant and changing the humidity, while the contrary (*RH* constant and *T* variable) could give misleading results since the *RH* would change correspondingly with *T*. In the paper of Salzmann et al. [8], we show that the hysteresis between swelling and deswelling under humidity largely depends on the heating/cooling rate while also affecting the detection of the lower critical solution temperature (LCST), which changes for different temperature ramp profiles. This effect was ascribed to the non-thermal equilibrium, whose effects are stronger for the faster ramps. In an environmental chamber, such effects are negligible because the water vapors and the sample are in thermal equilibrium; therefore, a more reliable characterization can also be conducted as a function of the temperature.

**Figure 1:**
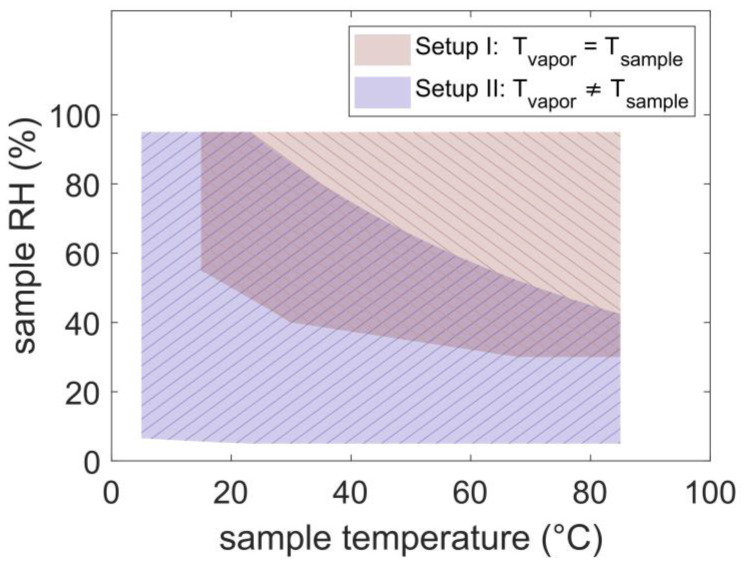
*RH*/temperature parameter space accessible with setup I (red area) and setup II (blue area). The parameter space for setup II was calculated considering *T*_vapor_ = 23 °C.

In this study, the sample was placed into an environmental chamber where it was in thermal equilibrium with the water vapor. A laser interferometer was used as a thickness detector: the laser pointed at the thin film sample, and the intensity of the reflected beam was collected via a photoresistor. The main difference to the traditionally used technique is that the sample and the environment have the same temperature, which widens the parameter space to the operating limits of the environmental chamber utilized. The parameter space of the two setups used within this study is depicted in Figure 1; setup I: environmental chamber, here *T*_vapor_ = *T*_sample_, and setup II: a hot plate and a humidity supplier, here *T*_vapor_ ≠ *T*_sample_. In setup I, the red area indicates the environmental chamber parameter space (from the datasheet). In setup II, the blue area depicts the relative humidity at the sample if the supplied vapor is at room temperature and has an *RH* between 5–95%. Below room temperature (23 °C), the *RH* at the sample increases above 100% and condensation would occur. Above room temperature, the *RH* at the sample decreases with temperature. At a temperature of 37 °C, which is an interesting temperature for body-related applications, the sample is only exposed to a relative humidity of 78%, and at 50 °C, only to 65%, further, at a temperature of 80 °C, a relative humidity of 45% can be accessed. Therefore, the reachable parameter space with setup II is limited, especially at elevated temperatures. While on the contrary, the parameter space of setup I fills exactly the missing area, where at an elevated temperature, the relative humidity values could reach up to 95%. 

The scope of the present paper is to critically discuss the window of applicability of the measurement methods using thermal gradients or in thermal equilibrium.

The two setups were tested on a thermo-responsive polymer thin film (poly N-vinylcaprolactam-co-di(ethylene glycol), divinyl ether, pNVCL-co-DEGDVE) deposited via initiated chemical vapor deposition on flat Silicon wafers and on Silicon wafers coated with ZnO. A comparison of thermo-responsiveness to a previously characterized setup with a hot plate and a humidified air supply [13,14], measured with spectroscopic ellipsometry, is performed to highlight the advantages and drawbacks of both techniques.

## 2. Materials and Methods

### 2.1. Setup I: Environmental Chamber Equipped with a Custom-Built Laser Interferometer (T_vapor_ = T_sample_)

The environmental chamber used for this setup was an Espec SH222, with an interior volume of 22 L (30 × 25 × 30 cm). With this machine, a relative humidity of 55% at 15 °C, 45 % at room temperature, and 30% at >70 °C can be accessed (see Figure 1, red area). 

**Figure 2:**
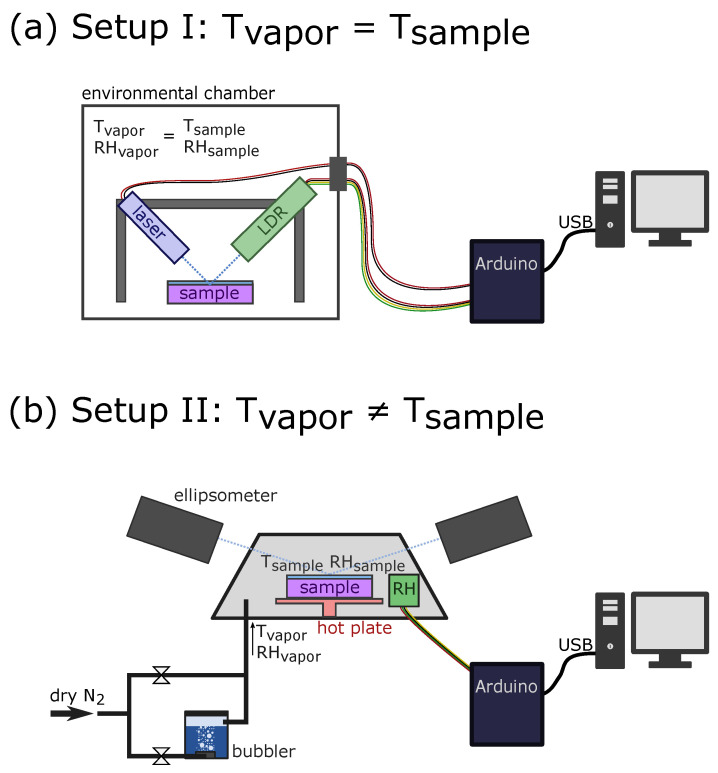
(**a**) Experimental setup of the custom-built interferometer placed inside the environmental chamber and operated by an Arduino from the outside. The blue laser illuminates the sample, and the reflected beam is measured by a photoresistor. (**b**) Setup with thermal gradients, where the sample is placed on a hot plate. The relative humidity is introduced via a bubbler system adjustable via two needle valves and tracked inside the chamber by a humidity sensor.

The chamber has a fit-through for cables for contacting devices from the inside to the outside. In this experiment, this was used in order to connect a blue laser (405 nm, Picotronic, Koblenz, Germany) as well as a photoresistor sensor module (WayinTop, China, the photoresistor was purchased already soldered on a PCB developer board, ready to plug and play) from the inside to an Arduino microcontroller on the outside, as depicted in Figure 2a. The photoresistor was shielded from any other light interference by an aluminum ring placed around the sensor and letting only the reflected beam illuminate the photoresistor. The laser was mounted on an aluminum frame (Makerbeam, Utrecht, Netherlands) and was pointing under an angle of 18° (with respect to the perpendicular) onto the sample. The photoresistor, mounted on the frame, was aimed at the refracted beam. The analog read-out signal of the photoresistor module was tracked each second. The read-out was a value between 0 V (bright light) and 5 V (dark), which is converted to a signal value of 0 to 1024, respectively. This signal was calibrated to obtain the film thickness.

For the temperature and humidity measurements, the relative humidity inside the chamber was raised stepwise in the range of 30–95% for four different temperature levels (10, 23, 35, and 50 °C). The targeted humidity/ temperature setpoint was held for 20 s (with limits of ± 1% RH and ± 0.2 °C) to provide a soaking time and ensure converged swelling. After that, the next humidity/temperature setpoint was ramped too. 

### 2.2. Setup II: Spectroscopic Ellipsometry with a Custom-Built Humidity Generator (T_vapor_ ≠ T_sample_)

The second approach was based on heating the sample on a hot plate and exposing it to humidified air, as schematized in Figure 2b. The thickness was tracked by a spectroscopic ellipsometer (M-2000V, J.A. Woollam, Lincoln, NE, USA), and the sample was placed on a domed heating stage (Linkam Heated Stage THMS600, J.A. Woollam, Lincoln, NE, USA). The heated stage increases the temperature of only the sample but not of the cell, which results in different temperatures for the sample surface and chamber environment. The humidity supply was accomplished by a custom-built bubbler system. Dry nitrogen is guided into a bubbler filled with distilled water. The humidified nitrogen is collected from the bubbler and mixed together with dry nitrogen through a second line before entering the heated stage. The relative humidity is manually regulated via two needle valves (Swagelok, Solon, OH, USA), controlling the direct dry nitrogen flow and the humidified nitrogen flow into the bubbler. A humidity and temperature sensor (SHT15, Sensirion, Switzerland) operated via an Arduino UNO is placed inside the heated stage to monitor the conditions inside. The sample temperature was set at the heating stage to 10, 23, 35, and 50 °C. In each temperature step, the humidity was raised gradually from 0 to 80%, tracked by the relative humidity sensor, while the ellipsometer measured a full spectrum (wavelength range 370–1000 nm), each for 4 s. Data fitting was performed by the software EASE Complete. The dry sample was modeled with three layers: a silicon bottom layer, a native oxide layer (1.7 nm thick), and a Cauchy layer, representing the optical transparent hydrogel. When the sample was exposed to humidified air, the Cauchy layer was exchanged by an effective media approximation model, which describes the refractive index of the layer as a combination (Bruggemann mixing method) of the dry hydrogel together with air with respect to thickness variation, as it was already performed in previous studies [6].

### 2.3. Preparation of Smart Hydrogel Thin Film Samples 

The chemicals used within this study are N-vinylcaprolactam (NVCL, Merck, Darmstadt, Germany), di(ethylene glycol), divinyl ether (DEGDVE, Merck, Darmstadt, Germany), di-tert-butylperoxid (TBPO, Merck, Darmstadt, Germany), and Diethylzinc (DEZ, Sigma Aldrich, St. Louis, MI, USA).

Thin film smart hydrogel films made of pNVCL-co-DEGDVE on a native silicon wafer (Siegert Wafer, Aachen, Germany) were fabricated via initiated chemical vapor deposition (iCVD) in a custom-built reactor. The inner diameter of the chamber was 100 mm. A two-stage rotary vane pump (DUO 20, Pfeiffer Vacuum, Aßlar, Germany) was used to maintain the reactor under vacuum. The chamber pressure was monitored via a capacitance manometer (600 Series, MKS Instruments, Andover, USA) and regulated via a throttle valve (MKS Instruments, Andover, MA, USA). The filaments were resistively heated by a low voltage power supply (PTN 350-5, Heinzinger, Germany). The temperature of the aluminum sample stage was controlled via the recirculating chiller (Polar Series 500 LC, Thermo Scientific, Waltham, MA, USA). The monomers NVCL and DEGDVE were heated to 85 and 70 °C, respectively, and entered the chamber with a flow of 0.1 ± 0.05 sccm and 0.2 ± 0.10 sccm, respectively. Prior to them entering the chamber, the vapors were pre-mixed in a reduced volume created by a metal plate with 1mm holes. The nominal DEGDVE amount was 42% [6]. The TBPO was kept at room temperature and entered with a flow of 0.9 ± 0.05 sccm. The substrate and filament temperatures were set to 30 and 200 °C, respectively. The deposition was carried out with a working pressure of 350 mTorr. The growth of the layer thickness was tracked in situ via laser interferometry (HeNe-Laser 633 nm, Thorlabs, Newton, MA, USA). The film produced had a thickness of 69 ± 1 nm, measured via spectroscopic ellipsometry.

The hydrogel swelling was also measured on a zinc oxide thin film. ZnO was deposited in a custom-built plasma atomic layer deposition (ALD) reactor [15]. During the plasma exposure (step A), the working pressure was controlled manually via a gate valve to 200 µbar. The plasma step was carried out in oxygen with a flow rate of 20 sscm, a plasma power of 60 W, and a duration of 10 s. The metalorganic precursor DEZ was fed into the reactor (step B) after the plasma exposure with a duration of 0.15 s. Nitrogen (flow rate of 20 sccm) was used to purge gas after the plasma treatment in step A and after the DEZ introduction in step B with a duration of 15 and 15 s, respectively. The complete deposition was carried out by the cyclic repeating step A and step B as follows: (A B)_n_A, with n as the cycle number, which was set to 200. The last step of the deposition was a plasma step to ensure the removal of the organic ligands of DEZ and to promote better adhesion of the hydrogel due to the surface activation. 

## 3. Results and Discussion

### 3.1. Simulation of Thin Film Interference

Thin film interference is a well-known physical phenomenon based on the superposition of light waves reflected at the surface and interfaces. One possible mathematical formalism, which is also used in this study, is known as the transfer matrix method and is well described in the book, Fundamentals of Photonic Crystal Guiding by M. Skorobogatiy and J. Yang [16]. Here, it is used to investigate the intensity of a blue laser beam reflected from a hydrogel layer coated on a native silicon wafer. In the formalism, if light penetrates into layered materials, each interface can be represented as a transfer matrix. 

For the transverse electric polarization (with *j* = the index of layer, *kz* = the z component of the wave vector, d = layer thickness), the transfer matrix from one to the next layer looks like:

(1) $$M_{j-1,j}^{TE}=\frac{1}{2}\left(\begin{array}{cc} \left(1+\frac{k_{z}^{j-1}}{k_{z}^{j}}\right)e^{ik_{z}^{j-1}d_{j-1}} & \left(1-\frac{k_{z}^{j-1}}{k_{z}^{j}}\right)e^{-ik_{z}^{j-1}d_{j-1}}\\ \left(1-\frac{k_{z}^{j-1}}{k_{z}^{j}}\right)e^{ik_{z}^{j-1}d_{j-1}} & \left(1+\frac{k_{z}^{j-1}}{k_{z}^{j}}\right)e^{-ik_{z}^{j-1}d_{j-1}} \end{array}\right)\;$$

And for the transverse magnetic polarization (with *ε* = the dielectric constant):

(2) $$M_{j-1,j}^{TM}=\frac{1}{2}\left(\begin{array}{cc} \left(1+\frac{k_{z}^{j-1}\epsilon_{j}}{k_{z}^{j}\epsilon_{j-1}}\right)e^{ik_{z}^{j-1}d_{j-1}} & \left(1-\frac{k_{z}^{j-1}\epsilon_{j}}{k_{z}^{j}\epsilon_{j-1}}\right)e^{-ik_{z}^{j-1}d_{j-1}}\\ \left(1-\frac{k_{z}^{j-1}\epsilon_{j}}{k_{z}^{j}\epsilon_{j-1}}\right)e^{ik_{z}^{j-1}d_{j-1}} & \left(1+\frac{k_{z}^{j-1}\epsilon_{j}}{k_{z}^{j}\epsilon_{j-1}}\right)e^{-ik_{z}^{j-1}d_{j-1}} \end{array}\right)\;$$

The complete system is described by applying these matrices in the correct order onto the vector of the expansion coefficients of the electric field, which are the wave amplitudes of forward and backward propagating beams. The amplitude of the reflected beam simplifies to be minus the relation of the two elements of the multiplied transfer matrices. In addition, the intensity of the reflected beam is the squared absolute value of this amplitude.

**Figure 3:**
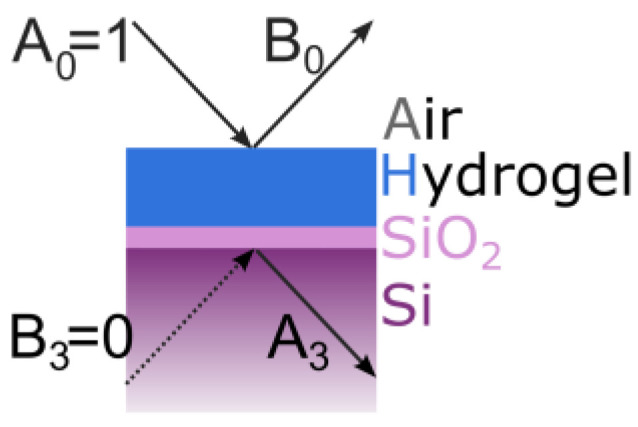
Schematic of the hydrogel layer on the silicon wafer. Arrows indicating the light beam. The A and B are the expansion coefficient of the electromagnetic wave propagating in the forward and backward direction in each layer.

In Figure 3, the cross-section of the sample is illustrated with the silicon on the bottom, the native silicon oxide in the middle, the hydrogel on top, and the air above. The forward and backward propagating beams are represented as arrows with the expansion coefficient *A*_0_, the incoming beam (set to 1), *B*_0_, the reflected beam, *B*_3_, the theoretical illumination from the backside, which is 0, and *A*_3_, the theoretical transmission. At each interface, the transfer matrices *M* act sequentially on the expansion coefficients (*A*_0_ and *B*_0_), which results in the expansion coefficients (*A*_3_ and *B*_3_).

(3) $$\left(\begin{array}{c} A_{3}\\ 0 \end{array}\right)=M_{{\mathrm{SiO}}_{2},\mathrm{Si}}M_{\mathrm{hydrogel},{\mathrm{SiO}}_{2}}M_{\mathrm{air},\mathrm{hydrogel}}\left(\begin{array}{c} 1\\ B_{0} \end{array}\right)\;$$

(4) $$\left(\begin{array}{c} A_{3}\\ 0 \end{array}\right)=\left(\begin{array}{cc} m_{11} & m_{12}\\ m_{21} & m_{22} \end{array}\right)\left(\begin{array}{c} 1\\ B_{0} \end{array}\right)\;$$

(5) $$B_{0}=-\frac{m_{21}}{m_{22}}\;$$

The intensity is the sum of the amplitude of the reflected beam (*B*_0_) for the transverse electric (*TE*) and the transverse magnetic (*TM*) component.

(6) $$I={\left|B_{0}^{TE}\right|}^{2}+{\left|B_{0}^{TM}\right|}^{2}\;$$

Within this study the input parameters (Table 1) were set to represent the custom-built interferometer and the sample: 

In the simulation, the refractive index is either a constant (*n*_dry hydrogel_) or a linear combination of the dry hydrogel and water (where *d* = the film thickness of the swollen hydrogel):

(7) $$n_{\mathrm{swollen}}=\frac{n_{\mathrm{dry}\;\mathrm{hydrogel}}\cdot d_{\mathrm{dry}\;\mathrm{hydrogel}}+n_{\mathrm{water}}\cdot (d-d_{\mathrm{dry}\;\mathrm{hydrogel}})}{d}$$

This approximation does not perfectly suit hydrogel’s swelling. Muralter et al. demonstrated a refractive index of p(NVCL) dependency on the *RH* and also on the swelling, which increased (from 1.520 to 1.528) when changing the *RH* from 0 to 37% (swelling of 4%), and attributed it to water only substituting the air in the polymer matrix voids, while swelling above 4%, the incorporated water expanded the matrix [6]. Nevertheless, the used linear combination should be interpreted as an initial assessment to give the reader a rough estimation of the influence of a refractive index responding to *RH*. 

Calculation of the *RH* if the sample and the environment are not at the same temperature.

The relative humidity (in %) is the relation between the partial pressure and the saturation pressure. The saturation pressure (*p*_sat_) at a certain temperature (*T*) can be calculated with the help of the Arden-Buck equation: [17]

(8) $$p_{\mathrm{sat}}=0.61121\;\cdot exp\left(\frac{17.368\;\cdot T}{T+238.88}\right)$$

In a closed setup, where spot C and spot D are at different temperatures, and a different *p*_sat_ value is also present, the relative humidity at spot C can be calculated via the product of the relative humidity and *p*_sat_ at spot D and the ratio of *p*_sat_ at spot C.

(9) $$RH_{C}=\frac{RH_{D}\cdot p_{\mathrm{sat},D}}{p_{\mathrm{sat},C}}$$

With these equations, it is possible to calculate the relative humidity at a cooled or heated sample, which is exposed to an environment at room temperature with a certain *RH*, which was shown as well by Muralter et al. [6]. Often, other studies are reported where the temperature is varied, and the *RH* is presumed to stay constant, while evidently, a change in temperature changes the *RH* drastically. For example, if the temperature drops from 20 to 19 °C, the *RH* increases by 5.6% [6]. During experiments where the sample is cooled, the environmental *RH* needs to be set to values below 95%, calculated by the Arden-Buck Equation (8), to prevent condensation [17].

### 3.2. Calibration of the Custom-Built Interferometer Setup (Setup I)

**Figure 4:**
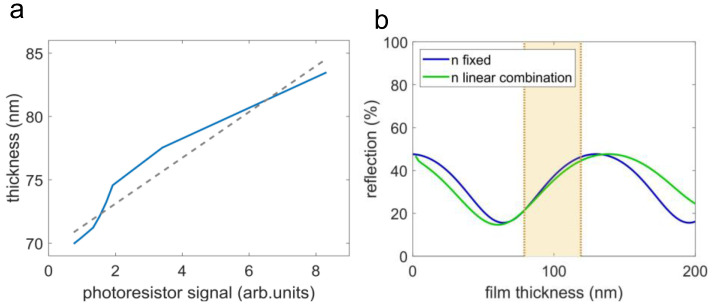
(**a**) Calibration curve for linking the photoresistor signal to the hydrogel’s thin film thickness. The solid line represents the experimental data and the dashed line represents a linear regression (*R*^2^ = 0.95). (**b**) The simulated intensity of the reflected beam versus the hydrogel’s film thickness. In blue: the refractive index of the hydrogel does not vary with the thickness (n fixed). In green: The thickness of the hydrogel is seen as a combination of hydrogel and water, and the refractive index is a linear combination of the refractive index of the dry hydrogel and water (n linear combination).

The first part of this study focuses on linking the signal of the custom-built interferometer to the thin film thickness, to obtain a calibration curve for further measurements. For that purpose, in the first place, the thickness of a p(NVCL-co-DEGDVE) thin film sample was measured via spectroscopic ellipsometry at different RH values at room temperature (23 °C), which acted as a reference curve. In the environmental chamber, the photoresistor signal was collected on the same sample at the same humidity levels as the previous experiment and again at room temperature. In Figure 4a, the calibration curve that links the hydrogel’s thin film thickness, obtained via spectroscopic ellipsometry, and the photoresistor signal of the custom-built interferometer, is plotted. Apparently, within this parameter region, linear regression is possible (*R*^2^ of 0.95). This trend is also confirmed by the simulation. 

In Figure 4b, the reflected intensity versus the hydrogel thickness is plotted with either the refractive index of the dry hydrogel or with a refractive index that is a linear combination of the dry hydrogel and water. In both cases, the intensity is a non-invertible function throughout the film thickness. However, in the region of the thickness variation of the hydrogel (highlighted area), the relation is invertible and close to a linear trend. Samples with a larger thickness variation may face the problem of a non-invertible calibration curve. Additionally, especially for higher thickness variations, the influence of a responsive reflective index may be more relevant, which might be caused by a larger water incorporation, thermal expansion, or a phase transition. The utilized calibration routine does not take such responsive refractive indices into account. However, for the hydrogel presented in this study, the linear regression model is suited well, and the influence of a responsive refractive index seems marginal in the highlighted area.

To identify the humidity and temperature responses with the interferometer setup, an experiment with a plane wafer was performed, where no response of the sample to temperature and humidity can be assumed. The result shows a slight temperature response attributable to either the laser or the photoresistor. No humidity-induced response could be spotted. For further measurements on hydrogels, the setup-related temperature response was subtracted.

### 3.3. Swelling Measurements 

**Figure 5:**
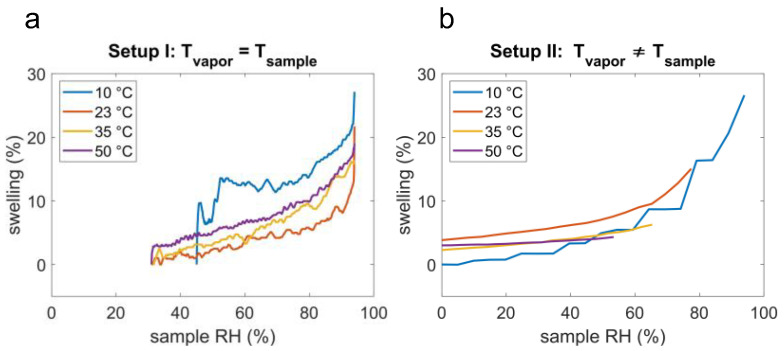
Swelling of the temperature responsive hydrogel at different temperatures and humidity levels, performed with (**a**) setup I, the custom-built interferometer within the environmental chamber, where *T*_vapor_ = *T*_sample_, or (**b**) setup II, the spectroscopic ellipsometry, where *T*_vapor_ ≠ *T*_sample_.

In Figure 5a, the swelling of the hydrogel measured with setup I is plotted in the *RH* range of 35–95% at temperatures of 10, 23, 35, and 50 °C. The sample thickness increases with increasing relative humidity at all temperature values, as expected. At approximately 78% RH, a change in the slope of the swelling curves is observable at all temperatures, as also shown by Muralter et al. [6]. Typically, at lower humidity levels, hydrogels form a glassy skin on the surface [18], where the hydrophobic polymer backbone faces the environment, and the impact of the humidity on the swelling is only minimal. At a certain relative humidity level, the hydrophilic functional groups turn outwards, the interaction of hydrogel and humidity increases, and a change in *RH* causes a pronounced swelling. This was also shown in a previous study on poly(hydroxyethyl methacrylate) (pHEMA), where the transition was observed at 75% *RH* [19].

The p(NVCL-co-DEGDVE) is a temperature-responsive hydrogel with a cross-linker amount dependent LCST in the range of 22 to 40 °C, measured in water [20]. The DEGDVE fraction in the hydrogel was 42%, which as Muralter et al. demonstrated, results in an LCST of (28 ± 4) °C [20]. At temperatures below the LCST, the swollen hydrophilic state can be expected, while above, the shrunken hydrophobic state is present. The data presented exhibits the largest swelling at temperatures of 10 °C, which is below the LCST, in nearly the complete relative humidity range, reaching 94% *RH*, the highest swelling of this measurement series of 27.19%. At temperatures above 10 °C, the sample swelling is less pronounced, reflecting the excellent expected response of a temperature-responsive hydrogel at temperature values above the LCST.

In Figure 5b, the swelling obtained via spectroscopic ellipsometry (setup II), in the presence of thermal gradients, is plotted versus the relative humidity of the sample surface calculated with the Arden-Buck Equation (8) in the range of 0–95%. Again, with rising relative humidity, an increase in swelling at all temperatures can be observed. For a sample temperature of 10, 23, 35, and 50 °C and incoming water vapor of 23 °C and 78% *RH*, the max humidity reached at the sample surface was calculated to be 94, 77, 65, and 53%, which resulted in a thickness increase of 27, 15, 6, and 4%, respectively.

The results of the measurements obtained by setup I and setup II already clearly visualize the different reachable sample *RH*/temperature parameter space of the two methods. With setup I, the interferometer inside the environmental chamber Espec SH222 (*T*_vapor_ = *T*_sample_) has only measurements above 30% *RH* that are accessible. On the contrary, with setup II, the hot plate with the humidity supply (*T*_vapor_ ≠ *T*_sample_) and with increasing sample temperatures, the maximum *RH* drops and already at 35 °C, the heated sample was never actually exposed to a relative humidity above the phase transition of hydrogels (≈75% *RH*), where the surface changes from a glassy skin to a hydrophilic one. This parameter space, which is of great importance for sensor material or functional contact lenses, cannot be accessed in the presence of thermal gradients.

**Figure 6:**
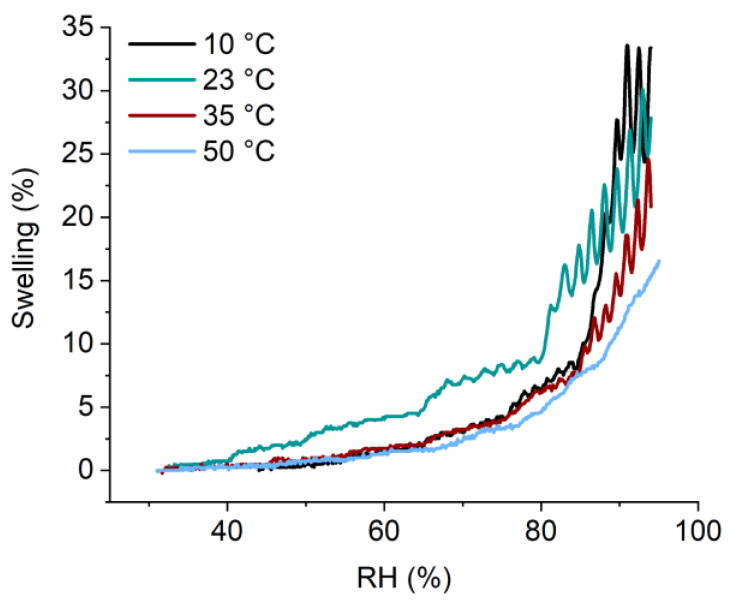
Swelling of the temperature responsive hydrogel at different temperatures and humidity levels, performed with setup I. The hydrogel was deposited on ZnO/Si.

To further prove our point, another case study is presented in Figure 6. Figure 6 showed the swelling measured on the same hydrogel as Figure 5 with a thickness of 151.24 nm, but deposited on a different substrate: on top of a 50 nm thick ZnO layer deposited on a native silicon wafer. The investigation of the thermo-responsiveness of pNVCL-co-DEGDVE was also tested on a ZnO thin film as the combination of these two materials has shown promise for multi-responsive sensors [15].

A calibration curve was performed in the same manner as previously described. In Figure 6, the swelling of the hydrogel is plotted at 10, 23, 35, and 50 °C in the relative humidity range of 30 to 95%. The obtained swelling curves of the hydrogel on Si show similar behavior to when the hydrogel is deposited on ZnO/Si. Again, with increasing humidity, the hydrogel’s swelling increases, reaching a 95% *RH*; a swelling of 33.38, 27.86, 20.83, and 16.57%; and temperature values of 10, 23, 35, and 50 °C, respectively.

## 4. Conclusions

In this study, humidity swelling under thermal equilibrium was studied using, as a case study, a hydrogel’s thin film response to temperature and humidity. The measurement was performed by interferometry, which is the placement of the sample and the detector in an environmental chamber. A comparison to the previous approach, where the sample and the humid air were not in thermal equilibrium, was demonstrated. The rationale behind this study is that in the presence of thermal gradients, the relative humidity measured in the environment does not represent the relative humidity at the sample surface. The use of an environmental chamber instead allows one to make more reliable assessments of the relative humidity at the sample surface, leading to an unequivocal interpretation of the swelling data. The relative humidity and temperature values can be controlled in the chamber where the sample is placed, and the thickness of the smart hydrogels can be measured. Especially for samples exposed to elevated temperatures (between room temperature and 80 °C), relative humidity levels of 95% are reachable. More specifically: the reachable RH/temperature parameter space is only limited by the used environmental chamber.

Beyond proposing a new method for the measurement of humidity and temperature-dependent swelling, the novelty of the paper is also attributed to the use of the interferometer for the detection of thickness changes in situ. More commonly, interferometry is used for precipitation or dissolution studies [21].

The drawback of using an interferometer at a single wavelength is that a calibration for each sample is needed. Further, the correlation between the thickness and the interferometer signal is theoretically not an invertible function and is sometimes refractive-index dependent. Nevertheless, the proposed method is an easily achievable extension for environmental chambers, which provides the possibility of measuring smart hydrogel’s thin film evolution in a previously unreachable *RH*/temperature parameter space.

## Figures and Tables

**Table 1:** Input parameters.

Incident Angle	Wavelength (nm)	*n*_air_	*d*_air_ (cm)	*n*_dry_hydrogel_	*d*_dry_hydrogel_ (nm)	*n*_SiO2@405nm_	*d*_SiO2_ (nm)	*n*_Si@405nm_
18°	405	1	2.6	1.540	69	1.745	1.7	5.46

## Data Availability

The data presented in this study are available upon request from the corresponding author.
